# Contribution of Shellfish Consumption to Lower Mercury Health Risk for Residents in Northern Jiaozhou Bay, China

**DOI:** 10.1155/2015/159521

**Published:** 2015-05-25

**Authors:** Lei Zhang, Lei Zhang

**Affiliations:** College of Resource and Environment, Qingdao Agricultural University, Changcheng Road 700, Qingdao 266109, China

## Abstract

Fish and marine mammal consumption are an important pathway for human exposure to mercury. The low mercury content in shellfish poses a low mercury health risk to people who consume shellfish. The objectives of this study are to detect mercury concentrations in different species of shellfish and to calculate the mercury health risk from shellfish consumption among traditional residents near northern Jiaozhou Bay. A total of 356 shellfish samples, which comprised 7 species from 5 different places in northern Jiaozhou Bay, were collected from April to June in 2012. The average mercury content in the collected shellfish ranged from 0.024 mg·kg^−1^ to 0.452 mg·kg^−1^. A total of 44 shellfish samples (12.36%) had mercury levels exceeding the national pollution-free aquatic products limit (0.3 mg·kg^−1^). Generally, the viscus had the highest mercury content among all parts of the shellfish. A positive correlation between mercury content and total weight/edible part weight was found in most species of the collected shellfish. The results showed that shellfish consumption resulted in the lower risk of mercury exposure to residents based on the calculation of daily intake (DI) and target hazard quotient (THQ).

## 1. Introduction

The high level of mercury (Hg) and its compounds, which are poisonous global pollutants, are an issue of international concern [1]. Elemental, inorganic, and organic mercury, which are mainly in the form of methylmercury (MeHg), harm the environment and human health. A recent breakthrough in toxicology research on Hg indicated that the population considered safe for Hg exposure before would become the subgroup in danger of health risks from Hg exposure. Total Hg is cardiovascular, visual, and nervous system toxicity substance, and it can cause health risk to human body even under the lower Hg dose [2–4]. Moreover, the myocardial infarction, atherosclerosis, and the damage on cardiovascular system also can be caused by low-dose MeHg exposure [5, 6].

Some studies conducted in the past decades have proven that consumption of fish products and marine mammals and rice grown in contaminated paddy fields or in mining areas is the primary pathway of Hg and MeHg exposure to the general population and subpopulations living in inland areas of China, respectively [7–13]. Furthermore, some papers have investigated that the low Hg levels exist in shellfish [14–20], which suggested that the shellfish consumption may be not an important pathway to the general human population in terms of Hg exposure.

In China, shellfish aquaculture is an important component of mariculture, and in 2006, the national mariculture production reached 14.456 million tonnes, including shellfish aquaculture production of 11.136 million tonnes, which accounted for about 77% of mariculture production for that year [21]. Jiaozhou Bay is an important shellfish-breeding base in north China. The bay has more than 30 years of shellfish breeding history. Benthic aquaculture for* Ruditapes philippinarum* and suspended aquaculture for* Argopecten irradians *are the main shellfish breeding patterns in the bay. In 2006, the production of* Ruditapes philippinarum* in Jiaozhou Bay reached 300000 t [22]. Higher levels of Hg were then found in the intertidal zone of Jiaozhou Bay, with the Hg pollution greater in the east coast intertidal ecosystem than in intertidal zone of the west bank [23]. Traditional and frequent shellfish consumption of local residents expose them to the possible effects of Hg on their health. Therefore, investigating the Hg content of different shellfish samples obtained from Jiaozhou Bay and assessing the Hg health risk posed by the exposure route of shellfish eating for the local population are necessary and important.

Limited literature on the subject shows that fish consumption is not an important Hg exposure pathway for coastal residents in Qingdao City, including Jiaozhou Bay [24], which is contrary to the previous conclusion from the other researches. For the traditional residents near Jiaozhou Bay, they have the habit of shellfish-eating every day, and the amount of shellfish consumption per day is more, which suggested that the shellfish-eating habit, not the fish-eating, seemed to be the Hg exposure pathway to the traditional residents in the investigated region. The conclusion in this reference “that the subpopulations living in the district of Jimo and Licang, in which the traditional residents live, had the higher hair Hg concentrations than that in the subgroups in the district of Chenyang and Laoshan, in which the foreign residents live,” seemed to support this opinion. Whether the habit of shellfish-eating causes Hg exposure risk to the residents near Jiaozhou Bay? To expand the literature, this study aims to determine the distribution of Hg content in shellfish samples among different species obtained from various locations in Jiaozhou Bay and to assess the Hg health risk resulting from the traditional shellfish consumption of coastal residents near Jiaozhou Bay. Hg content in the different parts of the shellfish and the relationship between Hg content and total weight/edible part weight of the shellfish are analyzed in this study. The results of this study provide a new understanding of Hg exposure route for residents living in the northern coastal areas in China.

## 2. Materials and Methods

### 2.1. Sample Collection

**Figure 1:**
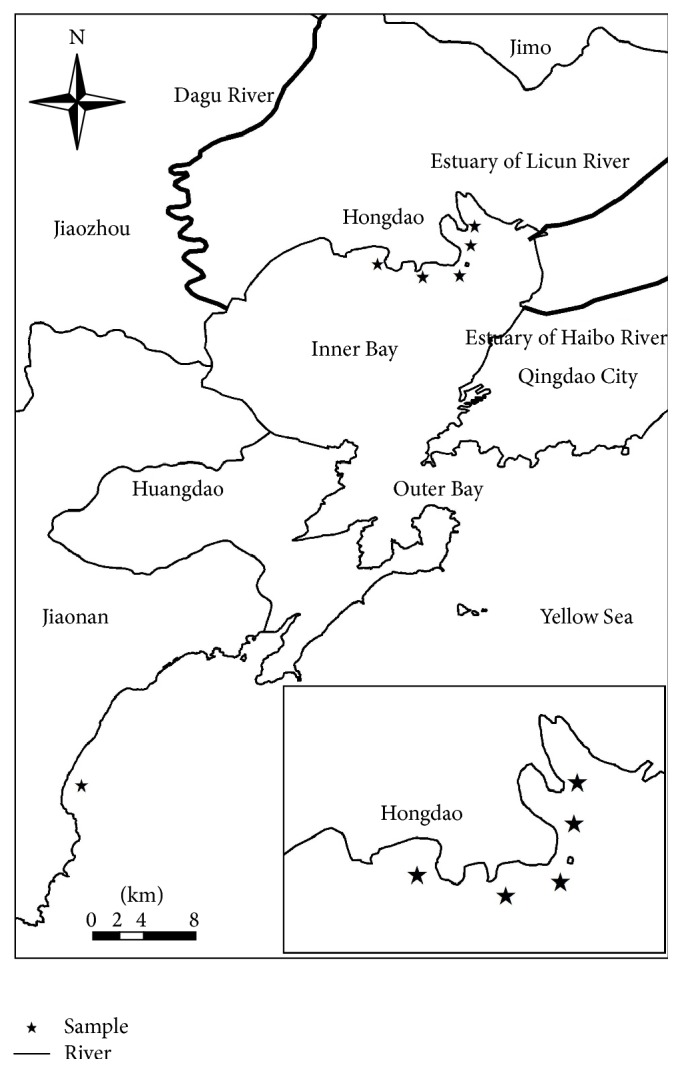
The spatial location for shellfish sampling in Jiaozhou Bay.

Jiaozhou Bay is located south of Shandong Peninsula in Shandong Province, northern China. From April to June in 2012, a total of 356 shellfish samples of 7 species were collected, which included* Ruditapes philippinarum* (*n* = 156, among which 101 samples collected from fishermen and 55 samples collected from remarket),* Haliotis asinina* (*n* = 21, all the samples gathered form remarket),* Busycon canaliculatum* (*n* = 38, among which 22 samples collected from fishermen and 16 samples collected from remarket),* Neptunea cumingi* (*n* = 27, among which 17 samples collected from fishermen and 10 samples collected from remarket),* Concha Ostreae* (*n* = 47, among which 35 samples collected from fishermen and 12 samples collected from remarket),* Sinonovacula* constricta (*n* = 36, among which 23 samples collected from fishermen and 13 samples collected from remarket), and* Argopecten irradians* (*n* = 31, among which 21 samples collected from fishermen and 10 samples collected from remarket), from five places in northern Jiaozhou Bay, specifically Dongdayang, Xidayang, Suliu Dock, Shaogezhuang, Houhan, and another sampling place, namely, Jiaonan, which was outside Jiaozhou Bay (Figure 1). When we arrived at the sampling places, we collected the shellfish samples from the fishermen or at the market, with simultaneous investigation of the original location of the shellfish source. At the time of the collection, the shellfish samples were kept alive and stored in “clean” seawater to remove sediments in their digestive system, with the time of shellfish samples at the remarked before purchase being less than one day. This step aimed at retaining the delicious taste of the shellfish, which was valued by the fishermen. And then, the shellfish samples with ice placed in labelled bags were stored in foam box during their transport to the lab to keep the samples fresh. At the lab, the shellfish samples were stored in icebox at −18°C for two weeks before their detection.

### 2.2. Sample Preparation and Hg Determination

Shellfish samples were removed from their shells, flushed with deionized water, and blotted by using filter paper. The edible part of the shellfish was homogenized and placed into labelled bags in an icebox prior to the measurement of the Hg content. For total Hg (THg) measurement, shellfish samples (0.5–1.0 g) were digested in a sand bath by using HNO_3_ and H_2_SO_4_ (4 : 1, v/v) catalysed by V_2_O_5_ and then detected by means of cold atomic absorption spectrophotometry by using an F732–V cold atomic absorption instrument and following the standard method in China [25].

The method measured by F732–V cold atomic absorption instrument has a measuring range of 0 to 10.0 *μ*g·L^−1^ and a sensitivity of not less than 0.05 *μ*g·L^−1^. All chemicals used in this experiment were produced by Sinopharm Chemical Reagent Co. Ltd., and all glassware were dipped in a mixture of HNO_3_ : H_2_O (1 : 3, v/v) for a whole night to eliminate the interference of ions attached to the glass walls. In a batch of 20 samples, three bland experiments and double parallel experiments for two shellfish samples, with their error range of less than 5%, were measured. The determination of reference materials for biological ingredients (GBW10050 (GSB–28)) was used to control the accuracy of the shellfish sample testing. Certified reference shrimp tissue (GBW–10050), with a certified value for Hg (0.049 ± 0.008 mg·kg^−1^) issued by Reference Material Information Center of China, was used to check the performance of analytical procedure. The recovery rate in this experiment ranged from 96.3% to 103.7%.

### 2.3. Calculation of Daily Intake (DI) and Target Hazard Quotient (THQ)

Hg intake from shellfish consumption was calculated using

(1) $$\begin{array}{c} DI=FIR\times C, \end{array}$$

where DI is Hg intake (*μ*g·d^−1^) and FIR is the daily intake rate for differential food (g·d^−1^). The average national daily intake of fish and shellfish consumption is 30.1 g·d^−1^ for adults (18 to 70 years old), 13.9 g·d^−1^ for teenagers (13 to 17 years old), and 15.4 g·d^−1^ for children (1 to 12 years old) in China [26]. *C* is the Hg concentration in the shellfish samples. In this experiment, the three shellfish species, such as* Haliotis asinine*,* Busycon canaliculatum*, and* Neptunea cumingi*, used this national recommended data mentioned above in the calculation process.

A variety of shellfish is available in the market near Jiaozhou Bay. Hence, traditional residents near Jiaozhou Bay consume the cheap shellfish for meals. Our survey, which was administered near Jiaozhou Bay, asked for information on the frequency and the daily amount of shellfish consumed by residents living near Jiaozhou Bay and the price of different species of shellfish production. The results indicated that the shellfish uptake ratio was different from that mentioned above.

The average daily intakes for the four kinds of shellfish samples, such as* Ruditapes philippinarum*,* Argopecten irradians*,* Sinonovacula constricta*, and* Concha Ostreae*, were set at 50 g·d^−1^ for adults, 23.09 g·d^−1^ for teenagers, and 25.1 g·d^−1^ for children on the basic of questionnaire investigation due to their large consumption per day and the cheap price. DIs for people of different ages were calculated by using the mean, minimum, and maximum Hg concentrations for the different types of shellfish gathered in this experiment.

The target hazard quotient (THQ) is a method that measures human health risk from pollutant exposure [27]. THQ hypothesizes that the absorbed dose of heavy metal is equal to the intake dose and its evaluative criterion is the ratio of intake dose to reference dose. No significant health risk to the population from pollutant exposure was determined when THQ is below 1. Otherwise, the health risk exists from pollutant exposure. THQ is calculated according to

(2) $$\begin{array}{c} THQ=\frac{\left(EF\times ED\times FIR\times C\right)\times 10^{-3}}{\left(RfD\times W_{AB}\times AT\right)}, \end{array}$$

where EF is exposure frequency (365 d·a^−1^), ED is exposure duration (45 years for adults, 15 years for teenagers, and 7 years for children), and FIR is the daily intake rate for differential food (g·d^−1^, same as above). *C* is the Hg concentration in the shellfish samples. RfD is the reference dose (*μ*g·kg^−1^ bw·d^−1^), with its PTWI value set by WHO (1972) to 0.714 *μ*g·kg^−1^ bw·day^−1^. *W*_AB_ is the average human body weight, and it considered a weight of 60 kg for adults and teenagers and 32.7 kg for children. AT is the average time (noncarcinogenic effects AT = ED × 365 d·a^−1^). ANOVA was used to compare the significant difference between the mean THg values of different species of shellfish and of different parts of the same shellfish sample.

## 3. Results

### 3.1. Distribution of Hg Concentration in Different Species of Shellfish

Table 1 lists the Hg content of the different species of shellfish samples collected from northern Jiaozhou Bay. Among the samples, the maximum Hg value appeared in* Argopecten irradians* and* Busycon canaliculatum* at 1.072 mg·kg^−1^ and 1.096 mg·kg^−1^, respectively. The* Ruditapes philippinarum* and* Busycon canaliculatum* had the maximum range of Hg distribution, with their maximum Hg levels being 940-fold and 365-fold higher than the minimum, respectively. A significant difference existed in the mean Hg value of the different shellfish species, which was remarked in Table 1. The results of all the kinds of shellfish had 2 groups, one with mean concentrations not exceeding 0.1 mg/kg and other with values greater than 0.24 mg·kg^−1^, with* Argopecten irradians* having the highest mean (0.452 mg·kg^−1^).

### 3.2. Comparison of Hg Content in Different Parts of Shellfish

*Argopecten irradians* shows significant differences in Hg contents between the different parts studied (Table 2). The mean Hg value of the viscus in* Argopecten irradians* was remarkably higher than that of the edible part, foot, and mantle (*p* < 0.01). The average Hg level in the edible part was significantly higher than that in the foot and mantle (*p* < 0.01). For* Haliotis asinine*, the average Hg value in viscus was observably higher than that in edible part (*p* < 0.05).

### 3.3. Relationship between Hg Content in Shellfish and Their Weight

Table 3 indicates the correlation between Hg contents in shellfish samples and their total weigh and edible part weight.* Ruditapes philippinarum* and* Neptunea cumingi* showed significant positive relationships between Hg contents in shellfish and their total weight and edible part weight (*p* < 0.05).* Haliotis asinina* and* Busycon canaliculatum* indicated remarkably significant positive relationship between Hg concentrations in shellfish and their total weight and edible part weight (*p* < 0.01). Inversely, the residual species of shellfish samples did not show any correlation between the Hg content in shellfish samples and their total weight or edible part weight.

### 3.4. Intakes and THQ of Hg through Shellfish Consumption

Table 4 presents the DI of Hg based on the consumption of different species of shellfish by the traditional residents according to different age groups. The average DI for all species of shellfish samples was less than the daily Hg intake given by FAO/WHO, with its value being 40 *μ*g·d^−1^ [28]. The maximum and minimum DI appeared in the consumption of* Argopecten irradians* and* Ruditapes philippinarum* by the residents in all age groups, respectively. Only the maximum DI through* Argopecten irradians* consumption by the adults exceeded the recommended value mentioned above.

Table 5 shows the THQ of Hg through the consumption of different species of shellfish for the traditional residents of different age groups. Same as the results of the DI calculation, all of the THQ for all types of shellfish based on the average Hg concentrations of shellfish samples in this study did not exceed 1, which suggested a lower Hg exposure risk through shellfish consumption for all the different age subgroups. The highest and lowest THQ observed in* Argopecten irradians* and* Ruditapes philippinarum* agree with the results of the DI calculation. Although the daily Hg intake and exposure years of children were less than those of other age subgroups, the THQ of children for all species of shellfish had the higher value in all age groups. The law of THQ calculation was as follows: THQ in adults groups > that in children groups > that in teenagers groups.

## 4. Discussion

The Hg content in 12.36% of the shellfish samples in this study exceeded the standard limit for the Hg content in pollution-free aquatic products (0.3 mg·kg^−1^) in China [29]. Moreover, the maximum and average Hg content of the shellfish samples were 2.76- to 91.33-fold and 4.96 to 150.67 times higher than those of the shellfish collected in the coastal areas of the Fujian Province (0.002–0.064 mg·kg^−1^) and in Brazil (0.206–0.397 mg·kg^−1^), Italy (0.023–0.100 mg·kg^−1^), and Spain (0.003–0.019 mg·kg^−1^), respectively [15–20, 30, 31]. However, the average Hg content in shellfish in this experiment was only 1.48%–40.72% to that in other fishery products, such as tuna and swordfish collected in Japan (1.11–1.82 mg·kg^−1^) and Spain (0.470–0.540 mg·kg^−1^) [20, 26]. The over standard rate was obtained by* Argopecten irradians*,* Haliotis asinina*, and* Busycon canaliculatum*, which had standard ratios of 66.67%, 40%, and 29.41%, respectively.* Ruditapes philippinarum*, which was collected from four sampling places (Dongdayang, Jiaonan, Shaogezhuang, and Houhan), was the most widely distributed shellfish species among all samples. The analysis results of the significant difference in the average Hg content of* Ruditapes philippinarum* from the above four places show that the Hg content in* Ruditapes philippinarum* from Dongdayang was significantly higher than those from Houhan (*p* < 0.05) and Jiaonan. Jiaozhou Bay is a semiclosed gulf, with the average and maximum water depth being 7 m and 64 m, respectively. The slow exchange velocity of seawater in this bay may lead to the easy accumulation of pollutants in the sediment. The heavy metals in the sediments of Jiaozhou Bay come from terrestrial and marine dual sources. The increase in heavy metal concentration in the seawater of Jiaozhou Bay continuously results from the increase in terrigenous pollution sources and the rapid development of the local economy. In aquatic ecosystem, the sediments usually act as a sink and source for Hg, in which Hg rereleases into the surrounding water inflecting by the biological, physical, and chemical factors [32]. After Hg entered into an aquatic ecosystem, Hg transformed into MeHg affecting by the various types of anaerobic microorganisms in the sediments, such as sulfate reducing bacteria, iron reducing bacteria, and methanogenic bacteria [33, 34]. The ratio of MeHg/THg in sediments usually ranged 0.1–2.4%, with the special samples reaching to 10% in sediments from some lakes and wetland [35–38]. After heavy metals contaminate the sediments, the chemicals that are released back into the overly seawater under suitable conditions can cause secondary pollution [39]. The growing shellfish in the sediments absorbs heavy metals by filter-feeding food, seawater, and direct contact with the surrounding sediments. This action may be the reason why the Hg content in the shellfish from the northern Jiaozhou Bay exceeded the Hg level in other places.

In this experiment, the mean Hg content in* Argopecten irradians* was remarkably higher than that in the residual shellfish. Compared with other bivalves, such as* Ruditapes philippinarum*,* Sinonovacula constricta*, and* Neptunea cumingi*, the larger size of* Argopecten irradians* resulted in higher Hg accumulation in their bodies, which also agreed by fish, namely, the size or age of fish, and their trophic position significantly affected the biomagnification of Hg in fish [40–42]. For* Argopecten irradians*, the organic detritus and plankton are their important food. Compared with water, the eating habit is the important pathway of Hg exposure for the organism in the aquatic ecosystem [43]. The chemical speciation of Hg may be an important factor inflecting Hg uptake for the aquatic organisms. In the part of Hg uptake in the organisms, HgCl_2_ and CH_3_HgCl were considered as the main species for Hg accumulation of Hg(II) and MeHg, respectively [44]. Moreover, the particulate matter is a transfer mechanism of Hg in water column to the sediment in an aquatic ecosystem [45]. Hg, major in MeHg, can biomagnify along the food chain, including the plankton, in an aquatic ecosystem [46, 47]. The planktonic organisms provide the essential nutrients to the higher trophic organisms, and at the same time, the pollutant, such as Hg, also is conveyed to them. And then, MeHg content in plankton increased with the increase of their size [48]. The dissolved organic carbon (DOC) was an important factor affecting the higher MeHg/THg ratio in water than that in periphyton, flocculent material and soil in everglades [49]. The inorganic Hg and monomethylmercury were strongly tied to organic matters, which is the important food for the shellfish, such as* Argopecten irradians*. When the organic matter content in sediment and water were rich, the amount of Hg accumulation in benthic invertebrates was lower and it seemed that monomethylmercury had the higher uptake rate to the benthic organism than inorganic Hg [50]. For shellfish samples, their Hg content in viscus was significantly higher than that in edible part (Table 2), which resulted from the filter action by gill and storage in viscus. For the benthic organisms living in the sediment, the factors for Hg accumulation in their bodies are complex.

*Haliotis asinine* has a longer life period of about 1 to 2 years, which could result in the Hg distribution in their edible part and shell equably. This quality could be explained partly by the positive correlation between Hg values and their edible part weight and total weight (Table 3). Generally, the proportion of the edible part to total weight increases gradually along with the age growth during the culture cycle of 1 to 3 years, with the growth speed of the edible part faster than that of the shell. For the Bivalves, such as* Argopecten irradians* and* Concha Ostreae*, they have a shorter aquaculture period of about six to eight months, and the larger weight of the shell compared with its edible part may be the reason for the noncorrelation between Hg content in the edible part and its total weight/edible part weight. For shellfish, such as* Argopecten irradians*, food residue in the gestive gland belonging to the viscus may be the main reason for the higher Hg content in this part than that in other parts.

In this study, two methods, namely, DI, and THQ, were used to estimate the Hg intake through shellfish consumption of the traditional residents of different age groups near Jiaozhou Bay. The calculation of DI was only based on the data of the daily Hg intake and Hg content in shellfish. Compared with Hg intake from fish and shellfish for Japanese people (24.28 *μ*g per capita per day) [26], Hg intake from shellfish samples in this experiment was 2.28–93.08% to that data mentioned above. Only for the average Hg intake from the consumption of* Argopecten irradians*, it was close to Hg intake from fish and shellfish from Japan. If we consider a weight of 60 kg for adults and a PTWI for total Hg of 5 *μ*g·kg^−1^ bw·week^−1^ (0.714 *μ*g·kg^−1^ bw·day^−1^), we have a DI of 42.84 *μ*g·day^−1^ for adults and teenagers. For children, considering a weight of 32.7 kg, it will be 23.35 *μ*g·day^−1^. The results of calculation of DI showed that the average Hg intake per capita per day was 1.29–52.75% and 2.58–48.59% for the subgroup of adults/teenagers and children, respectively. The fact indicated that there was a lower Hg health risk from shellfish product for all the residents, including the sensitive crowd children, living near Jiaozhou Bay. However,* Argopecten irradians* captured in this experiment presenting the highest Hg level in all the shellfish samples also had the highest daily Hg intake, which was 1.15-fold higher than the corresponding data for children. In the long term, it seems to be safe for the residents (including children) from Hg exposure through shellfish consumption basic on the kinds and producing areas in this experiment, which also agreed by the result of the calculation of THQ. The outcome of THQ suggested that the result of THQ was 1.3–52.7% and 2.6–48.6% to the standard value of 1 for the subgroup of adults/teenagers and children, respectively. For all the subgroup, the results of the two calculation methods suggested that the long period of consumption for all kinds of shellfish in this experiment is safe for health risk from Hg intake. However, the long term consumption of* Ruditapes philippinarum* and* Argopecten irradians* seems to have a slightly higher Hg health risk to the subgroups of adults and children due to their maximum Hg concentrations. Therefore, the children, as the sensitive subpopulaiton, should reduce their shellfish-eating amount for the safe of Hg intake during a long period.

## 5. Conclusions

The Hg concentration of 12.43% found in the shellfish samples exceeded the national limit for pollution-free aquatic products. The Hg distribution in shellfish had a difference among these species of shellfish. The traditional residents near Jiaozhou Bay had low Hg intake through shellfish consumption based on the DI and THQ calculation. And then, these residents, for all the different age subpopulations, had lower Hg intake through shellfish consumption according to the estimation from the two methods.

## Figures and Tables

**Table 1:** Hg contents in the different species of shellfish samples (wet weight, mg·kg^−1^).

Species	Number	Range	Average	Geometric mean	S.D.
*Argopecten irradians *	31	0.147–1.072	0.452a	0.381	0.280
*Busycon canaliculatum *	38	0.003–1.096	0.250b	0.104	0.186
*Haliotis asinina *	21	0.085–0.477	0.243b	0.208	0.134
*Concha Ostreae *	47	0.023–0.256	0.095c	0.073	0.068
*Sinonovacula constricta *	36	0.033–0.171	0.080c	0.068	0.048
*Neptunea cumingi *	27	0.006–0.201	0.073c	0.054	0.053
*Ruditapes philippinarum *	156	0.001–0.094	0.024cd	0.016	0.015

Notes: there is no significant difference between those containing same letters, and there is significant difference between those containing different letters, with *α* = 0.05.

**Table 2:** The distribution of Hg in the different parts of shellfish samples (wet weight, mg·kg^−1^).

Species	Name of part	Average	Range	S.D.	Number
*Argopecten irradians *	Viscus	0.839a	0.523–1.072	0.177	31
	Edible part	0.526b	0.174–0.871	0.273	31
	Mantle	0.296bc	0.155–0.612	0.171	31
	Foot	0.156bc	0.147–0.171	0.008	31

*Concha Ostreae *	Edible part	0.096a	0.037–0.256	0.067	47
	mantle	0.047a	0.023–0.124	0.035	47

*Busycon canaliculatum *	Viscus	0.251a	0.011–1.096	0.233	38
	Edible part	0.231a	0.003–0.947	0.219	38

*Haliotis asinina *	Viscus	0.250a	0.091–0.477	0.028	21
	Edible part	0.121b	0.085–0.398	0.097	21

Notes: there is no significant difference between those containing same letters, and there is significant difference between those containing different letters, with *α* = 0.05.

**Table 3:** Correlation between Hg content in different types of shellfish and their total weight/edible part weight.

Species	Correlation coefficient (*r*)	Equation	Pearson coefficient (*P*)	Number
*Ruditapes philippinarum* (total weight)	0.252^*∗*^	*y* = 0.00194*x* + 0.0147	0.027	156
*Ruditapes philippinarum* (edible part weight)	0.323^*∗*^	*y* = 0.00689*x* + 0.01223	0.004	156
*Argopecten irradians* (total weight)	0.452	*y* = 0.0221*x* − 0.0221	0.141	31
*Argopecten irradians* (edible part weight)	0.386	*y* = 0.0351*x* + 0.0852	0.215	31
*Sinonovacula constricta*(total weight)	0.480	*y* = 0.0072*x* + 0.0011	0.161	36
*Sinonovacula constricta*(edible part weight)	0.294	*y* = 0.0063*x* + 0.0428	0.409	36
*Haliotis asinina* (total weight)	0.938^*∗∗*^	*y* = 0.01672*x* − 0.25966	0.000	21
*Haliotis asinina* (edible part weight)	0.948^*∗∗*^	*y* = 0.02183*x* − 0.16669	0.000	21
*Busycon canaliculatum* (total weight)	0.908^*∗∗*^	*y* = 0.00728*x* − 0.05267	0.000	38
*Busycon canaliculatum* (edible part weight)	0.925^*∗∗*^	*y* = 0.01552*x* − 0.01723	0.000	38
*Concha Ostreae* (total weight)	0.381	*y* = 0.0019*x* + 0.0216	0.073	47
*Concha Ostreae* (edible part weight)	0.378	*y* = 0.0082*x* + 0.0317	0.076	47
*Neptunea cumingi * (total weight)	0.671^*∗*^	*y* = 0.00267*x* − 0.03605	0.024	27
*Neptunea cumingi * (edible part weight)	0.693^*∗*^	*y* = 0.0092*x* − 0.00839	0.018	27

^*∗∗*^Remarkably significant correlation; ^*∗*^significant correlation.

**Table 4:** Hg intake to residents of different age groups near northern Jiaozhou Bay by shellfish consumption values (*μ*g·d^−1^).

Species	The daily Hg intake through shellfish consumption for residents of different age					
	Adults		Teenagers		Children	
	Average	Range	Average	Range	Average	Range
*Ruditapes philippinarum *	1.200	0.050–47	0.554	0.023–21.705	0.602	0.025–23.594
*Argopecten irradians *	22.600	7.350–53.600	10.440	3.394–24.752	11.345	3.690–26.907
*Sinonovacula constricta *	4.000	1.650–8.550	1.847	0.762–3.948	2.008	0.828–4.292
*Haliotis asinina *	7.314	2.559–14.358	3.378	1.182–6.630	3.742	1.309–7.346
*Busycon canaliculatum *	7.525	0.090–32.990	3.475	0.042–15.234	3.850	0.046–16.878
*Concha Ostreae *	4.750	1.155–12.800	2.194	0.531–5.911	2.385	0.577–6.426
*Neptunea cumingi *	2.197	0.181–6.050	1.015	0.083–2.794	1.124	0.092–3.095

**Table 5:** THQ of Hg in the different species of shellfish from northern Jiaozhou Bay.

Species	THQ through shellfish consumption for residents of different age					
	Adults		Teenagers		Children	
	Average	Range	Average	Range	Average	Range
*Ruditapes philippinarum *	0.028	0.001–1.097	0.013	0.001–0.507	0.026	0.001–1.010
*Argopecten irradians *	0.527	0.172–1.251	0.244	0.079–0.572	0.486	0.158–1.152
*Sinonovacula constricta *	0.093	0.039–0.199	0.043	0.018–0.092	0.086	0.036–0.184
*Haliotis asinina *	0.170	0.060–0.334	0.079	0.028–0.155	0.160	0.056–0.315
*Busycon canaliculatum *	0.176	0.002–0.770	0.081	0.001–0.356	0.165	0.002–0.723
*Concha Ostreae *	0.111	0.027–0.299	0.051	0.012–0.138	0.102	0.025–0.275
*Neptunea cumingi *	0.051	0.005–0.010	0.024	0.002–0.065	0.048	0.004–0.133

## References

[1] Steenhuisen F., Wilson S. J. (2015). Identifying and characterizing major emission point sources as a basis for geospatial distribution of mercury emissions inventories. *Atmospheric Environment*.

[2] Lim S., Chung H.-U., Paek D. (2010). Low dose mercury and heart rate variability among community residents nearby to an industrial complex in Korea. *NeuroToxicology*.

[3] Beuter A., de Geoffroy A., Edwards R. (1999). Quantitative analysis of rapid pointing movements in Cree subjects exposed to mercury and in subjects with neurological deficits. *Environmental Research*.

[4] da Costa G. M., dos Anjos L. M., Souza G. S. (2008). Mercury toxicity in Amazon gold miners: visual dysfunction assessed by retinal and cortical electrophysiology. *Environmental Research*.

[5] Salonen J. T., Seppanen K., Nyyssonen K. (1995). Intake of mercury from fish, lipid peroxidation, and the risk of myocardial infarction and coronary, cardiovascular, and any death in Eastern Finnish men. *Circulation*.

[6] Salonen J. T., Seppänen K., Lakka T. A., Salonen R., Kaplan G. A. (2000). Mercury accumulation and accelerated progression of carotid atherosclerosis: a population-based prospective 4-year follow-up study in men in eastern Finland. *Atherosclerosis*.

[7] Bonsignore M., Manta D. S., Oliveri E. (2013). Mercury in fishes from Augusta Bay (southern Italy): risk assessment and health implication. *Food and Chemical Toxicology*.

[8] Shao D., Liang P., Kang Y. (2011). Mercury species of sediment and fish in freshwater fish ponds around the Pearl River Delta, PR China: human health risk assessment. *Chemosphere*.

[9] Zaza S., de Balogh K., Palmery M., Pastorelli A. A., Stacchini P. (2015). Human exposure in Italy to lead, cadmium and mercury through fish and seafood product consumption from Eastern Central Atlantic Fishing Area. *Journal of Food Composition and Analysis*.

[10] Chien L.-C., Gao C.-S., Lin H.-H. (2010). Hair mercury concentration and fish consumption: risk and perceptions of risk among women of childbearing age. *Environmental Research*.

[11] Li P., Feng X., Yuan X. (2012). Rice consumption contributes to low level methylmercury exposure in southern China. *Environment International*.

[12] Meng M., Li B., Shao J.-J. (2014). Accumulation of total mercury and methylmercury in rice plants collected from different mining areas in China. *Environmental Pollution*.

[13] Li B., Shi J. B., Wang X. (2013). Variations and constancy of mercury and methylmercury accumulation in rice grown at contaminated paddy field sites in three Provinces of China. *Environmental Pollution*.

[14] Groth E. (2010). Ranking the contributions of commercial fish and shellfish varieties to mercury exposure in the United States: implications for risk communication. *Environmental Research*.

[15] Li J., Zeng X. C., Huang Z. Y. (2012). A survey of mercury concentrations in shellfish and the risk assessment. *Journal of Chinese Institute of Food Science and Technology*.

[16] Li X.-Z. (2009). Total mercury content in seawater-cultured shellfish in Fujiam Province and its health risk assessment. *Chinese Journal of Ecology*.

[17] Wang Z. (2010). Preliminary analysis and evaluation on the environment of shellfish culture area and the quality of shellfishes in north-central coast of Fujian Province. *Journal of Fujian Fishers*.

[18] Weng X. (2006). The investigation of the cultivating shellfish quality in middle and Eastern part of Fujian. *Fujian Journal of Animal Husbandry and Veterinary medicine*.

[19] Lemos V. A., dos Santos L. O. (2014). A new method for preconcentration and determination of mercury in fish, shellfish and saliva by cold vapour atomic absorption spectrometry. *Food Chemistry*.

[20] Olmedo P., Pla A., Hernández A. F., Barbier F., Ayouni L., Gil F. (2013). Determination of toxic elements (mercury, cadmium, lead, tin and arsenic) in fish and shellfish samples. Risk assessment for the consumers. *Environment International*.

[21] Wei Q. S., Sun P. X., Xu Z. J., Wang Z. X., Zang J. Y. (2010). A research on shellfish aquaculture environment safety evaluation and monitoring system technology. *Ocean Development and Management*.

[22] Zhang M. L. (2008). *The research of carrying capacity of shellfish in the Jiaozhou Bay [Master thesis]*.

[23] Liu Y. F., Wu S. Y., Sun S. X., Sun Y. G. (2009). Comparison between sedimental contamination in intertidal zone of Jiaozhou Bay and the counterpart of Laizhou Bay. *Coastal Engineering*.

[24] Zhang L. (2009). Study on the human hair mercury levels in the residents living in the different function regions in Qingdao and influence factors. *Journal of Safety and Environment*.

[25] China EPA Soil quality—Determination of total mercury—Cold atomic absorption spectrophotometry.

[26] Nakagawa R., Yumita Y., Hiromoto M. (1997). Total mercury intake from fish and shellfish by Japanese people. *Chemosphere*.

[27] USEPA Risk based concentration table [EB/OL]. http://www.epa.gov/reg3hwmd/risk/human/index.htm.

[28] Ostapczuk P., Valenta P., Rutzel H., Nurnberg H. W. (1987). Application of differential pulse anodic stripping voltammetry to the determination of heavy metals in environmental samples. *Science of the Total Environment*.

[29] General Administration of Quality Supervision (2001). Agricultural product safety and quality pollution-free aquatic products safety requirements. *GB*.

[30] Bille L., Binato G., Cappa V. (2015). Lead, mercury and cadmium levels in edible marine molluscs and echinoderms from the Veneto Region (north-western Adriatic Sea—Italy). *Food Control*.

[31] Plessi M., Bertelli D., Monzani A. (2001). Mercury and selenium content in selected seafood. *Journal of Food Composition and Analysis*.

[32] Marvin C., Painter S., Rossmann R. (2004). Spatial and temporal patterns in mercury contamination in sediments of the Laurentian Great Lakes. *Environmental Research*.

[33] Avramescu M.-L., Yumvihoze E., Hintelmann H., Ridal J., Fortin D., Lean D. R. S. (2011). Biogeochemical factors influencing net mercury methylation in contaminated freshwater sediments from the St. Lawrence River in Cornwall, Ontario, Canada. *Science of the Total Environment*.

[34] Monperrus M., Tessier E., Point D. (2007). The biogeochemistry of mercury at the sediment-water interface in the Thau Lagoon. 2. Evaluation of mercury methylation potential in both surface sediment and the water column. *Estuarine, Coastal and Shelf Science*.

[35] Gilmour C. C., Henry E. A., Mitchell R. (1992). Sulfate stimulation of mercury methylation in freshwater sediments. *Environmental Science and Technology*.

[36] Kannan K., Smith R. G., Lee R. F. (1998). Distribution of total mercury and methyl mercury in water, sediment, and fish from South Florida estuaries. *Archives of Environmental Contamination and Toxicology*.

[37] Ullrich S. M., Tanton T. W., Abdrashitova S. A. (2001). Mercury in the aquatic environment: a review of factors affecting methylation. *Critical Reviews in Environmental Science and Technology*.

[38] Shi J.-B., Liang L.-N., Jiang G.-B., Jin X.-L. (2005). The speciation and bioavailability of mercury in sediments of Haihe River, China. *Environment International*.

[39] Wei T. J., Zhang H. L., Li T., Lu Y. H., Zhou N., Zhu M. X. (2012). Study of the geochemical forms of heavy metals in surface sediments of Jiaozhou Bay and Qingdao inshore areas. *Periodical of Ocean University of China*.

[40] McIntyre J. K., Beauchamp D. A. (2007). Age and trophic position dominate bioaccumulation of mercury and organochlorines in the food web of Lake Washington. *Science of the Total Environment*.

[41] Dušek L., Svobodová Z., Janoušková D. (2005). Bioaccumulation of mercury in muscle tissue of fish in the Elbe River (Czech Republic): multispecies monitoring study 1991–1996. *Ecotoxicology and Environmental Safety*.

[42] Taylor D. L., Kutil N. J., Malek A. J., Collie J. S. (2014). Mercury bioaccumulation in cartilaginous fishes from Southern New England coastal waters: contamination from a trophic ecology and human health perspective. *Marine Environmental Research*.

[43] Unrine J. M., Jagoe C. H., Brinton A. C., Brant H. A., Garvin N. T. (2005). Dietary mercury exposure and bioaccumulation in amphibian larvae inhabiting Carolina bay wetlands. *Environmental Pollution*.

[44] Laporte J. M., Truchot J. P., Ribeyre F., Boudou A. (1997). Combined effects of water pH and salinity on the bioaccumulation of inorganic mercury and methylmercury in the shore crab *Carcinus maenas*. *Marine Pollution Bulletin*.

[45] Guentzel J. L., Portilla E., Keith K. M., Keith E. O. (2007). Mercury transport and bioaccumulation in riverbank communities of the Alvarado Lagoon System, Veracruz State, Mexico. *Science of the Total Environment*.

[46] Guedes Seixas T., Moreira I., Siciliano S., Malm O., Kehrig H. A. (2014). Mercury and selenium in tropical marine plankton and their trophic successors. *Chemosphere*.

[47] Ciesielski T., Pastukhov M. V., Szefer P., Jenssen B. M. (2010). Bioaccumulation of mercury in the pelagic food chain of the Lake Baikal. *Chemosphere*.

[48] Kainz M., Telmer K., Mazumder A. (2006). Bioaccumulation patterns of methyl mercury and essential fatty acids in lacustrine planktonic food webs and fish. *Science of the Total Environment*.

[49] Liu G., Cai Y., Philippi T. (2008). Distribution of total and methylmercury in different ecosystem compartments in the Everglades: implications for mercury bioaccumulation. *Environmental Pollution*.

[50] Lawrence A. L., Mason R. P. (2001). Factors controlling the bioaccumulation of mercury and methylmercury by the estuarine amphipod *Leptocheirus plumulosus*. *Environmental Pollution*.

